# Assessing the Impact of Integrated Community-Based Management of Severe Wasting Programs in Conflict-Stricken South Sudan: A Multi-Dimensional Approach to Scalability of Nutrition Emergency Response Programs

**DOI:** 10.3390/ijerph18179113

**Published:** 2021-08-29

**Authors:** Andre M. N. Renzaho, Gilbert Dachi, Kibrom Tesfaselassie, Kiross Tefera Abebe, Ismail Kassim, Qutab Alam, Nawal Sadick Shaban, Tesfatsion Shiweredo, Hari Vinathan, Chandrakala Jaiswal, Hellen Martin Abraham, Khamisa Ayoub Miluwa, Rashidul Alam Mahumud, Eric Ategbo, Biram Ndiaye, Mohamed Ag Ayoya

**Affiliations:** 1 Translational Health Research Institute, School of Medicine, Western Sydney University, Sydney, NSW 2560, Australia; 2 Maternal, Child and Adolescent Health Program, Burnet Institute, Melbourne, VIC 3004, Australia; 3 UNICEF South Sudan, Totto Chan Compound, Juba P.O. Box 45, Sudan; gdachi@unicef.org (G.D.); ktesfaselassie@unicef.org (K.T.); kabebe@unicef.org (K.T.A.); ikassim@unicef.org (I.K.); qalam@unicef.org (Q.A.); nsshaban@unicef.org (N.S.S.); tshiweredo@unicef.org (T.S.); hvinathan@unicef.org (H.V.); cjaiswal@unicef.org (C.J.); hmabraham@unicef.org (H.M.A.); eaategbo@unicef.org (E.A.); 4 Nutrition Department, South Sudan Ministry of Health, Juba 81111, Sudan; khamisaayoub@yahoo.com; 5 NHMRC Clinical Trials Centre, Faculty of Medicine and Health, The University of Sydney, Camperdown, NSW 2006, Australia; rashed.mahumud@sydney.edu.au; 6 UNICEF Somalia, RA International Compound, Aden Adde International Airport Area, Mogadishu, Somalia; bindiaye@unicef.org (B.N.); mayoya@unicef.org (M.A.A.)

**Keywords:** South Sudan, community-based management of severe wasting, effectiveness of treatment, relapse

## Abstract

Community-based management of severe wasting (CMSW) programs have solely focused on exit outcome indicators, often omitting data on nutrition emergency preparedness and scalability. This study aimed to document good practices and generate evidence on the effectiveness and scalability of CMSW programs to guide future nutrition interventions in South Sudan. A total of 69 CMSW program implementation documents and policies were authenticated and retained for analysis, complemented with the analyses of aggregated secondary data obtained over five (2016–2020 for CMSW program performance) to six (wasting prevention) years (2014–2019). Findings suggest a strong and harmonised coordination of CMSW program implementation, facilitated timely and with quality care through an integrated and harmonised multi-agency and multidisciplinary approach. There were challenges to the institutionalisation and ownership of CMSW programs: a weak health system, fragile health budget that relied on external assistance, and limited opportunities for competency-based learning and knowledge transfer. Between 2014 and 2019, the prevalence of wasting fluctuated according to the agricultural cycle and remained above the emergency threshold of 15% during the July to August lean season. However, during the same period, under-five and crude mortality rates (10,000/day) declined respectively from 1.17 (95% confidence interval (CI): 0.91, 1.43) and 1.00 (95% CI: 0.75, 1.25) to 0.57 (95% CI: 0.38, 0.76) and 0.55 (95% CI: 0.39, 0.70). Both indicators remained below the emergency thresholds, hence suggesting that the emergency response was under control. Over a five-year period (2016–2020), a total of 1,105,546 children (52% girls, 48% boys) were admitted to CMSW programs. The five-year pooled performance indicators (mean [standard deviations]) was 86.4 (18.9%) for recovery, 2.1 (7.8%) for deaths, 5.2 (10.3%) for defaulting, 1.7 (5.7%) for non-recovery, 4.6 (13.5%) for medical transfers, 2.2 (4.7%) for relapse, 3.3 (15.0) g/kg/day for weight gain velocity, and 6.7 (3.7) weeks for the length of stay in the program. In conclusion, all key performance indicators, except the weight gain velocity, met or exceeded the Humanitarian Charter and Minimum Standards in Humanitarian Response. Our findings demonstrate the possibility of implementing robust and resilient CMSAM programs in protracted conflict environments, informed by global guidelines and protocols. They also depict challenges to institutionalisation and ownership.

## 1. Introduction

For decades, South Sudan has experienced armed conflicts, a pattern that continued after it gained independence from Sudan on 9 July 2011 [1,2]. With over 60 different major ethnic groups in a new nation with little political and economic experience, outbreaks of new armed civil conflicts post-independence escalated into conflicts between the various ethnic groups, leadership personalities centred on the struggle for political power and dominance, and armed factions [1,2]. Consequently, since its independence, the country has been characterised by weak state structures; stunted economic, social, and political progress; and mass population displacements [2,3,4]. These factors have led to the country’s inability to fully meet the basic human needs of its population. They have also impaired the country’s capacity to build social protection blocks, economic structures and political participation pathways that enable communities to improve and sustain their living conditions.

Despite improving and favourable harvests since 2015 [5,6], high inflation and political instability have contributed to the increase in food and transport costs, leading to alarming rates of food insecurity and child malnutrition. For example, data by the International Monetary Fund indicate that inflation, as measured by the annual consumer price index, increased from 1.7% in 2014 to 380% in 2016, then declining to 87.2% in 2019 and 29.7% in 2020 [7]. In addition, the 2019 FAO/WFP Crop and Food Security Assessment Mission [8] reported that food prices started to soar in July 2015 (i.e., before the currency collapse observed in 2016), and staple foods were among most affected commodities. When compared to levels of 12 months earlier, prices reported in December 2019 showed an increase of 75 to 90% for sorghum and maize grains, 45% for wheat flour, and 20% for cassava. As a consequence of these stressors, the 2021 global report on food crisis by the Global Network Against Food Crises in collaboration with Food Security Information Network estimated that nearly 7.2 million (60% of the population) have been experiencing food crisis [9]. Food crisis represents a situation where households are only marginally able to meet minimum food needs by depleting essential livelihood assets or through unhealthy-coping strategies, characterised by high or above-usual acute malnutrition prevalence due food consumption gaps [9]. The food crisis is a result of the confluence of insecurity, conflict-related displacements, and increasing food prices [9], and unhealthy coping strategies can be multidimensional. They may encompass the sale of assets, resorting to low quality or nutritionally inferior diets, limiting portion size or skipping meals to allow small children to eat, and sending household members away to work, beg, gather wild food, or hunt [10]. In addition, the report estimated that 108,000 people would experience food catastrophe throughout 2021 (i.e., an extreme lack of food and/or other basic needs, with evident starvation, death, destitution and extremely high levels of acute malnutrition) [9].

In many countries experiencing protracted armed conflicts, persistent wasting remains a serious public health challenge. Persistent wasting is used to define a situation in which wasting prevalence among children under five years remains consistently above the emergency threshold of 15% over several years, and in some cases, over decades [11]. This has been the case of South Sudan, where the annual wasting prevalence has remained above 15% since its independence, declining from 22.7% in 2010 to 16.2% in 2019 [12]. In South Sudan wasting prevalence trends are hugely impacted by seasonal variations. High wasting prevalence is often recorded during the lean season, with relatively low wasting prevalence during the harvest and post-harvest seasons. However, given that malnutrition causal pathways are complex, drivers of persistent wasting are difficult to pinpoint and involve an interplay of three underlying causes—food, care, and health; compounded by cultural, social, economic, and political determinants [11].

Focusing on the pattern of persistent wasting as a measure of success could fail to depict the effectiveness of nutrition interventions. Although the primary goal of humanitarian response in protracted armed conflicts is to reduce malnutrition-related morbidity, it is vital to include and monitor more sensitive indicators such as mortality [13]. Maintaining crude and under-five mortality rates at or below a rate double the pre-conflict baseline must provide a better insight into the emergency response’s effectiveness [13]. A pooled analysis of 10 prospective studies on associations of sub-optimal growth with all-cause and cause-specific mortality in children under five years of age found that severe wasting (weight-for-height z-score <−3 or oedema) and moderate wasting (weight-for-height z-score between −3 and −2) are associated with nine-fold and three-fold increased risk of child mortality, respectively [14].

One of the live-saving interventions widely used in emergency response to reduce excess, avoidable deaths and illness is the management of child wasting through emergency feeding programs [13] However, the management of severe wasting has evolved over the last four decades [15,16,17,18]. Early approaches were centre-based where children suffering from severe wasting were admitted to therapeutic feeding programs, paediatric wards or even nutrition rehabilitation units for weeks and treated according to the World Health Organization (WHO) protocols. Children admitted to the program had to be accompanied by one of the parents, keeping them away from the rest of the family. Several issues associated with centre-based feeding programs have been extensively documented [15,16,17,18]. They include low coverage rates, which lead to late presentation and associated complications and overcrowding and the risk of cross-infection. These centres often had limited capacity and lacked adequate skilled staff, leading to heavy staff workloads, high default rates, and possibly high-risk behaviours among mothers to cover meals [15,16,17,18].

To overcome the above challenges, community-based management of wasting programs were piloted in 2000 [19]. They were found to achieve better outcomes than traditional approaches [16,20,21] and were endorsed by the United Nations in 2007. They have since gained widespread acceptance in humanitarian and non-humanitarian contexts across low- and middle-income countries, many of which have developed national guidelines. These programs have two complementary treatment programs: community-based management of severe wasting (CMSW) programs and those addressing moderate wasting. This paper is only concerned with CMSW programs.

Significant investments have accompanied the widespread uptake of CMSW programs, spearheaded by government ministries of health and supported by United Nations (UN) agencies such as the United Nations Children’s Fund (UNICEF) in close collaboration with the World Food Programme (WFP), the World Health Organisation (WHO), and national and international NGOs. The three key characteristics of CMSW programs are (1) partnerships, outreach, and capacity-building driven mobilisation of community actors (e.g., governments, community structures and agents of change, and non-government organisations- NGOs); (2) inpatient or stabilisation centres for children affected by severe wasting with medical complications; and (3) outpatient therapeutic programs (OTP) for children affected by severe wasting without medical complications. The availability of ready to use therapeutic foods (RUTF) has made it possible to treat children in their homes.

Most studies reporting the impact of CMSW programs have focused on exit outcome indicators (e.g., the percentage of exiting children due to recovery, death, defaulting, or not recovering) and their enablers (e.g., weight gain velocity, length of stay in the programs, and feeding practices) [20,21,22,23]. However, such an approach does not do justice to the expanded impact of CMSW programs. Nutrition emergency preparedness (e.g., partnerships, outreach, and capacity-building driven mobilisation of community actors), and wasting prevention (e.g., training on infant and young child practices) and treatment (e.g., RUTF and systematic treatment on admission) go hand-in-hand at all stages of CMSW program implementation [11]. This integrated approach has been the cornerstone of the emergency response in South Sudan. Therefore, this study aimed to document good practices and generate evidence on the effectiveness and scalability of CMSW programs to guide future nutrition interventions in South Sudan

## 2. Materials and Methods

### 2.1. Data Source and Procedures

A multi-dimensional approach to scalability and integration was used to assess CMSW programs’ impact at all stages of implementation. It involved a desk review of CMSW program implementation (2014–2020), and the analysis of aggregated secondary data obtained over five years (2016–2020 for CMSW program performance), and nutrition emergency preparedness and wasting prevention strategies over six years (2014–2019). A desk review allowed us to gather existing documents and secondary data in a coherent and usable format to understand the planning, implementation, and evaluation of CMSW programs. The desk review involved scanning the literature (Supplementary Materials S1), complemented by the analysis of secondary data. The first stage of scanning of the literature involved identifying and reviewing common sources of information and all documents pertaining to OTP and stabilisation centres.

A team of 14 people (the researcher, a research fellow, 11 UNICEF staff, and 1 Ministry of Health, South Sudan staff) met to draw a matrix of documents to be gathered for analyses and their sources. The developed matrix identified the six criteria for inclusion in the analyses: (1) operational documents (describing how processes and activities are performed in all stabilisation centres and OTP), (2) CMSW program implementation plans/reports, (3) strategic plans (NGOs and UN agencies’ goals and action plans related to severe wasting prevention, treatment, and management), (4) monitoring and evaluation plans and reports, (5) national guidelines, and (6) field and assessment reports. Using this matrix, UNICEF staff collated the documents to be reviewed. The scanning of CMSW program documents was complemented by a quick scan of the Field Exchange tri-annual magazine and Google Scholar covering the period between 2014 and 2020 to retrieve additional documents that met any of the six criteria above. Since Google Scholar uses automatic stemming, the quick literature scan used the following combination of search terms without quotations to retrieve documents with word variations based on our keywords (as the use of quotation retrieves only the exact word) [24]: South Sudan AND (acute malnutrition OR severe acute malnutrition OR wasting OR severe wasting) AND (community-based management of malnutrition OR CMAM OR outpatient therapeutic program OR OTP OR inpatient care OR stabilisation centre). A total of 69 articles and documents were authenticated through organisational logos or publisher/bibliographic details were retained for analysis (Supplementary Materials S1). The study protocol and procedures were approved by Western Sydney University’s Human Research Ethics Committee of (HREC Approval Number: H14405).

The effectiveness and performance of CMSW programs were assessed using aggregated secondary data (a combination of individual-level data) obtained over six years (2014–2019), extracted from UNICEF South Sudan’s monitoring, evaluation, accountability and learning database. In South Sudan, the nutrition information management system has significantly evolved over time from time-hungry excel-based reporting systems prone to data inaccuracies to an application-based monitoring system. An application-based monitoring system allowed data synchronisation. It saved time, improved data quality and consistency, and was easily scalable. Timely access to data and real-time sharing of activities facilitated early and timely detection of ineffectiveness. The net result was a quick customised response to arising performance issues. However, challenges still existed. The reporting system was designed to only capture site-based aggregated nutrition program performance data. Due to increasing reporting pressure on implementing NGO partners and limited resources, aggregated information obtained from multiple centres did not include individual-level data. Hence, UNICEF used aggregated data from various partners to inform its nutrition programming strategies. The aggregated individual-related outcome data were averaged by implementing NGO partners, by geographic area, by year, and by type of CMSW programs.

The study used three sources of data: the standardised monitoring and assessment of relief and transitions (SMART) and food security and nutrition monitoring system (FSNMS) surveys, and CMSW programs’ performance data. Data to depict trends in child wasting were extracted from FSNMS surveys from rounds 14 to 25 (the period covered by the current study). A detailed methodological approach has been provided in these reports (see for example [12]). Briefly, the FSNMS surveys’ main objective was to provide regular updates on the food security and nutritional situation status. A total of 25 FSNMS surveys were completed by 2019 but our study focused on data from round 14 onward. FSNMS survey did not collect mortality data. Therefore, mortality data were collated from SMART surveys. Both FSNMS and SMART surveys used a two-stage cluster random sampling method. In the first stage, clusters were selected using a probability proportional to population size. Households were randomly selected at the second stage [12,25]. All data were collected by trained enumerators using electronic tablets and data collected uploaded onto a dedicated online server.

### 2.2. Study Outcomes

#### 2.2.1. CMSW Program Scalability

The retrieved documents were reviewed to characterise drivers of CMSW program scalability. The scalability focused on outlining scaling up pathways being implemented, including vertical and horizontal integration to support expansion, standardisation, and/or replication [26]. Vertical integration analysis focused on examining the extent to which two or more United Nations agencies, NGOs, government departments and ministries, and community organisations were collaborating in terms of pooling resources, sharing responsibilities, and strengthening and integrating capabilities and competencies to maximise CMSW programs’ performance. Horizontal integration focused on collaborations and inter-organisational relationships between agencies at the same level of capacity and responsibilities, such as between United Nations agencies or between international NGOs to elucidate internal systems and procedures as well as role delineation that facilitate CMSW program implementation capacity. Consistent with Milat et al. [26]’s scalability assessment tool, a total of 11 indicators were retained:

- Intervention delivery pathways and harmonisation of implementation plans;
- Delivery system (reach, adoption, and expansion);
- Provision of technical assistance and strong organisational capacity at all levels CMSW program implementation;
- Integrating approaches within government systems, policies, priorities, and targets;
- Generating and disseminating evidence to inform policy and programs;
- Engaging communities as co-designers and co-implementers (ownership);
- Using monitoring and evaluation systems to extract lessons learnt;
- Building an enabling environment and strengthening the decision support;
- Facilitating partnerships and integration;
- Delineation of roles and responsibilities;
- Funding and financial sustainability.

#### 2.2.2. Emergency Preparedness and Wasting Prevention

The retrieved documents were further reviewed to identify the critical aspects of nutrition emergency preparedness and severe wasting prevention that are incorporated into CMSW programs. Three broad indicators for nutrition preparedness were retained: (1) activities implemented to increase the community’s ability to prevent and better respond to wasting such as training on infant and young child feeding (IYCF) practices; (2) conducting integrated rapid response mechanism (IRRM) missions; and (3) carrying out evidence-based nutrition and anthropometric surveys to predict the burden of severe wasting and reach of services, assess capacity to respond, and put in place response mechanisms. Severe wasting prevention focused on assessing trends in the prevalence of wasting as well as the under-five mortality rate (U5MR), and crude mortality rate (CMR) and comparing them with emergency thresholds. The U5MR is a more sensitive indicator to changes in health status than the CMR, but the CMR is a critical and most useful indicator to both monitor and evaluate the severity of an emergency situation [13].

#### 2.2.3. CMSW Performance

The performance of OTPs and stabilisation centres was assessed by comparing CMSW program outcomes with SPHERE minimum standards [13]. However, whilst most of the studies evaluating the effectiveness of CMSW programs have tended to exclude medical transfers, hence inflating their estimate of recovery rates, there is emerging recognition of this vital indicator [27,28,29,30] and its inclusion in national guidelines [31]. In South Sudan, including medical transfers as a critical indicator is paramount because there are cases of children with severe wasting that are often referred from the CMSW programs to other health facilities for further medical investigation and care (that is, outside any nutrition program) [31]. Therefore, parameters for CMSW program performance included in this study were:

- Recovered: children discharged after a successful recovery. It is calculated as the number of children recovered/total number of discharged × 100;
- Died: children who died during treatment in CMSW programs. It is calculated as the number of deaths/total number of discharged × 100;
- Defaulted: children who did not complete treatment due to absenteeism (absent during three consecutive visits, defaulter confirmed at third absence). It is calculated as the number of defaulters/total number of discharged ×100;
- Medical transfers: children referred to hospital or health facility children outside nutritional programs for further medical investigation or medical treatment. It is calculated as the number of medical transfers/total number of discharged × 100;
- Not recovered: children who did not meet the discharge criteria for recovery after four months of treatment. It is calculated as the number of individuals not recovered/total number of discharged × 100;
- Relapse: children who completed treatment and discharged as “recovered” but developed severe wasting within a period of two months and got readmitted for further treatment. It is calculated as the number of relapse/total admission ×100;
- Weight gain velocity: calculated as weight gain (weight at discharge − weight at admission in grams)/(the weight on admission in Kilograms × length of stay in the program); expressed as g/kg/person/day;
- Length of stay: calculated as the date at discharge minus the date at admission, expressed in days.

### 2.3. Data Analysis

Content analysis of retrieved documents was undertaken using a deductive approach [32]. As indicated in the foregoing discussion, 11 indicators were pre-determined [33] and modelled on Milat et al. [21]’s scalability assessment tool. Content analysis of retained documents focused on looking at phrases correlated with these predetermined indicators [32,33]. We determined what was to be searched for a priori, then we documented occurrences of indicators and their operationalisation within the document. For the quantitative component, data were analysed using Stata version 14. Exploratory data analysis was carried out to check for normality and the effect of outliers. Outliers for weight gain velocity and length of stay were less than 0.78% and were excluded from the analysis because they returned implausible values. CMSW program outcomes (recovered, died, not recovered, defaulter, medical transfers, relapse, weight gain velocity, and length of stay) were tabulated and compared to international Sphere minimum standards [13]. Given that our outcome measures were continuous variables, in order to assess the effect of socio-demographic, weight gain velocity, and length of stay on t exit outcomes (i.e., reason for exiting the program) and relapse, a generalised linear model (GLM) was used. The GLM model produces more efficient and unbiased regression estimates when examining quantitative continuous outcome variables with non-normal distribution. It relaxes several assumptions of traditional linear regression models. In this study, two-tailed probability values of <0.05 were considered as the statistically significant level.

## 3. Results

### 3.1. CMSW Scalability

Drivers of the scalability and stabilisation centres and OTPs are summarised in Table 1. Overall, there was strong evidence of the integration of CMSW into national guidelines and robust monitoring, evaluation, accountability and learning systems to support evidence-based decision making. Clear intervention delivery systems and pathways as well as standardised treatment protocols existed. They were complemented by extended technical assistance, capacity building mechanisms for NGOs and health workers. There was a delineation of role and responsibilities to minimise duplication of efforts. For example, UNICEF oversaw all programs associated with the prevention, treatment, and management of severe wasting whilst WFP coordinated and managed all programs pertaining to the treatment and the management of moderate wasting. There were many strategies in place to strengthen this division of labour, including joint action plans and management meetings, mutually agreed governance structures and partnership commitments, joint coordination at the country and field level, and quarterly and annual progress reports. This delineation of roles and responsibilities was also reflected in their selection of implementing NGO partners. Strong partnerships and coordination among UN agencies and implementing NGO partners facilitated national capacity building, reduced the likelihood of siloed programs and parallel efforts, and strengthened the expanded decentralisation of CMSW programs. The existence of a Nutrition Cluster response plan helped build an enabling environment, strengthened coordination and decision support, facilitated partnerships and integration, and increased program expansion and coverage.

Data in Table 2 depict the expansion of CMSW programs. By 2020, CMSW programs operated in all 79 counties, experienced an exponential growth and scaled up coverage from about 351 operational nutrition sites in 2014 to 1171 nutrition sites in 2020.

Vitamin A supplementation programs expanded across counties whilst the number of nutrition education and capacity building activities increased significantly between 2014 and 2020 amid series of conflicts that have displaced one in three South Sudanese to neighbouring countries. The number of IYCF counselling sessions for caregivers of children aged 0–23 months surpassed planned targets for all years except 2018. UNICEF’s IRRM missions, SMART surveys, and standardised treatment protocols became the cornerstone of decision making and expansion of CMSW programs. IRRM missions and SMART surveys provided timely data to inform situational and nutrition analyses, predict risks, and address emergency response gaps. The screening of children to identify severely wasted children, the provision of maternal and child nutrition education activities, vitamin A supplementation, and preventive chemotherapy (deworming) were integral parts of IRRM missions. There were plans and structures for improving the implementation of evidence-based practice and information systems. This expansion was characterised by collaboration between implementing NGO partners and UN agencies at all levels of management, service delivery, and technical assistance.

However, there were serious challenges to engaging communities and government as co-designers and co-implementers (ownership) and funding and financial sustainability. Beyond community outreach activities and training of government and NGO health workers in IYCF and CMSW programs, there was no evidence of integrating key community members into the leadership roles governing CMSW programs. The number of well-trained cadres of community health workers and facility-based health staff of government actors who could adequately and independently implement and evaluate CMSW programs without external technical assistance was limited. In addition, there was a paucity of clear guidance on and a weak context-specific framework for community mobilisation and information systems pertaining to regular community-level screening for children affected by severe wasting, referrals for severe wasting treatment, and monitoring of relapses and medical transfers.

There was no evidence of community mobilisation strategies and action plans informed by community-led assessments. Although UN agencies and international NGOs made great strides in improving and harmonising assessment approaches, data collection procedures, and data quality, high staff turnover remained a threat to knowledge loss. Capacity-building activities focused on the nutrition information working group and the integrated food security phase classification technical working group. The groups’ membership did not include local communities as stakeholders. A weak context-specific framework for community mobilisation and information systems meant that the understanding of the community’s key players and community dynamics was limited, compounded by the ongoing political unrest, militancy, and insurgency. With the Nutrition Department of the Ministry of Health being severely resource-constrained, the government’s effective participation in collaborative decision-making processes was impaired. The operation of CMSW programs heavily relied on emergency funding and imported RUTF. This coupled with limited integration of CMSW programs into national health budgets and local health systems, further compromising government ownership of the programs.

### 3.2. Severe Wasting Prevention and Nutrition Emergency Preparedness

The treatment and management of severe wasting were complemented by prevention strategies and nutrition emergency preparedness. Severe wasting prevention focused on promoting IYCF practices through counselling caregivers of children aged 0–23 months, capacity building of implementing NGO partners and health workers in IYCF practices, and vitamin A supplementation and deworming (Table 2). Nutrition emergency preparedness and the response centred on expanding and scaling up OTPs, and IRRM missions and SMART surveys to inform the delivery of critical and lifesaving services to vulnerable people, including children. IRRM missions were broad and allowed lead agencies, together with implementation NGO partners, to provide food and nutrition programs with both preventive and curative aspects (e.g., general food distribution, nutrition supplies for management of wasting, deworming, and vitamin A supplementation). Through an emergency nutrition coordination platform and joint rapid assessments, IRRM missions enabled a situational and nutrition analysis to better understand risks and vulnerabilities. They informed preparedness plans and actions and provided needed data to gauge emergency response needs and required operational capacities. IRRM missions were also equally critical in informing early warning surveillance systems with clearly defined early warning indicators, timely response, and CMSW program expansion strategies. Their findings were embedded in all aspects of severe wasting prevention, treatment, and management.

**Figure 1:**
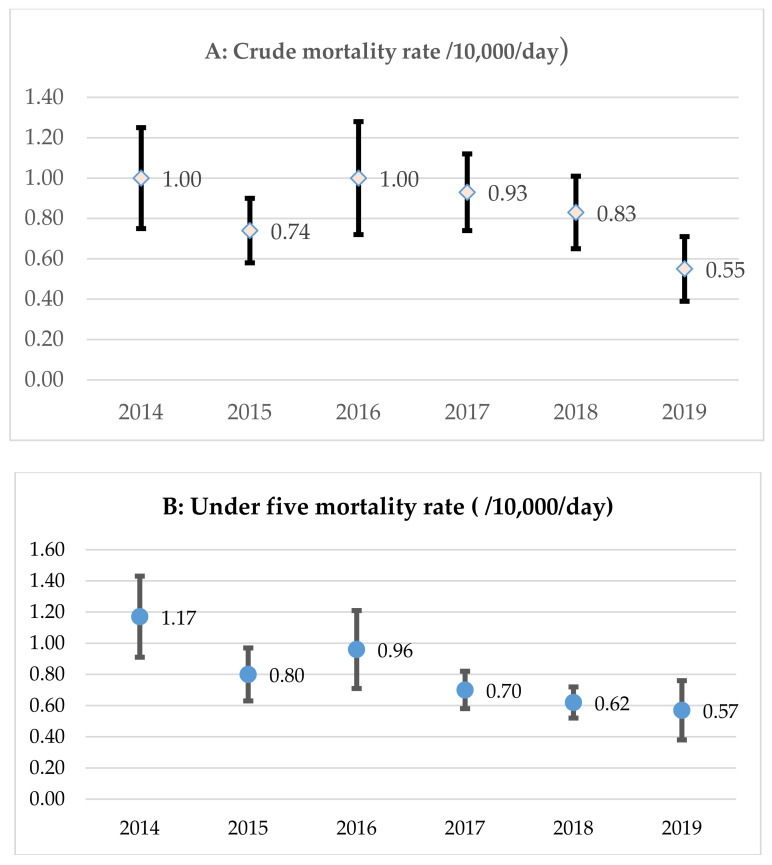
Trends in crude mortality and under five mortality rate.

**Figure 2:**
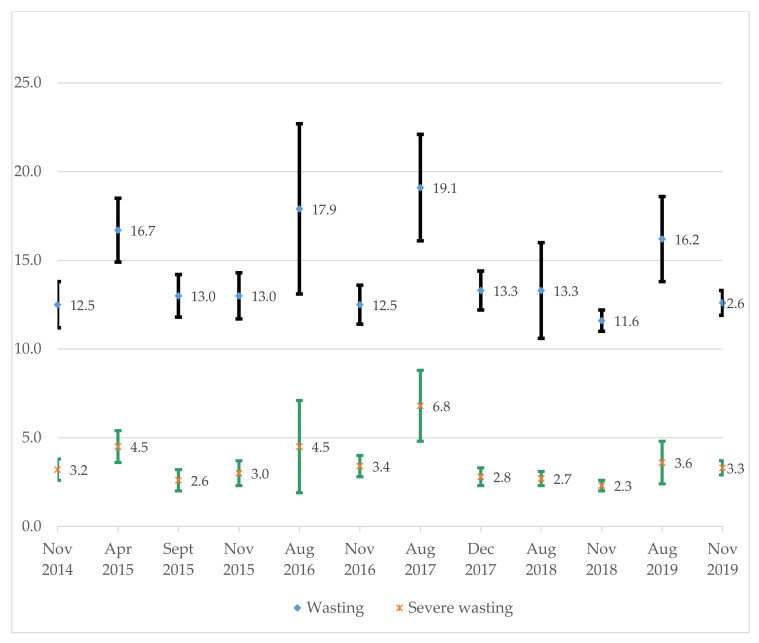
Trends in the prevalence of child wasting. Note: Data compiled from FSNMS surveys. Wasting = weight-for height < −2 Z-scores and/or bilateral oedema; Severe wasting = weight-for height < −3 Z-scores or bilateral oedema.

Figure 1 and Figure 2 provide trends in CMR, U5MR, and wasting prevalence. CRM declined significantly from 1.00 (95% confidence interval (CI): 0.75, 1.25) per 10,000 persons per day in 2014 to 0.55 (95% CI: 0.39, 0.70) in 2019. A similar trend was observed for U5MR, which declined from 1.17 (95% CI: 0.91, 1.43) per 10,000 live births per day in 2014 to 0.57 (95% CI: 0.38, 0.76) in 2019. Both CRM and U5MR remained below the emergency thresholds, suggesting that the emergency response was under control. However, between 2014 and 2019, the prevalence of wasting fluctuated according to the agricultural cycle. Nutrition surveys were often carried out twice a year: one survey during the lean season (i.e., the period of time between planting and harvesting when job opportunities were scarce and food stores were low; April to July) and another in the post-harvest period (October to December). Overall, wasting prevalence remained above the emergency threshold of 15% during the lean season, hence depicting a ‘very high’ public health concern of long-lasting nature.

### 3.3. Performance of CMSW

Relapse rate, exit outcomes (recovered, died, defaulted non recovered, and transferred), weight gain velocity, and length of stay in the programs are summarised in Table 3. Over a five-year period, a total of 1,105,546 children (52% girls, 48% boys) were admitted to CMSW programs. The analysis of exit outcomes found that 919,747 out of 1,105,546 admitted children (83.19) exited the programs. Of those exiting, 86.4% (*n* = 795,029) recovered after treatment, 2.1% (*n* = 19,039) died during treatment, 5.2% (*n* = 47,367) were defaulters after loss to follow-up, 1.7% (*n* = 15,912) were discharged after failing to recover, and 4.6% (*n* = 42,400) were transferred for further medical treatment.

The relapse rate was estimated at 2.2% whilst the length of stay in the program and weight gain velocity averaged 6.7 weeks and 3.3 g/kg/day, respectively. All key performance indicators, except the weight gain velocity, met or exceeded SPHERE minimum standards.

Table 4 shows unadjusted coefficients depicting factors associated with exit outcomes and relapse rate. Multivariate analyses are summarised in Table 5. The adjusted models show no difference between boys and girls, and the result did not vary by the child’s age. However, children whose sex was categorised as “other” were less likely to recover and more likely to die during treatment or transferred for medical treatment than girls. Further analyses indicated that children whose sex was categorised as “other” were more likely to be those aged 5 years or older. Although the target group for CMSW programs were children under the age of 5 years, the programs still admitted older children with severe wasting. Given that older children were not a priority and fell outside the target age group for CMSW programs, the likelihood of gender being misclassified among this group was higher. Overall, performance indicators improved significantly over time, with results for recovery and defaulter being better in 2019 and 2020 when compared to 2016. In addition, the rate for non-recovery was lower in 2020 than in 2016. However, over time the relapse rate remained significantly higher than the 2016 baseline.

The results also depict regional inequalities. Whilst the non-recovery rate did not vary by states, all states recorded a higher recovery rate except Northern Bahr el Ghazal and had a lower defaulter rate than Central Equatoria. In addition, compared to Central Equatoria, the proportion of children who died after admission was significantly lower in Lakes, Jonglei, Unity, and Warrap, whilst the proportion of medical transfers was lower in Eastern Equatoria, Lakes, Jonglei, and Western Bahr el Ghazal. The proportion of children relapsing was significantly higher in Northern Bahr el Ghazal and Warrap than in Central Equatoria. Length of stay in the program and weight gain velocity were not associated with exit outcomes and relapse. However, children admitted in OTPs recorded lower death and transfer rates but higher defaulter and non-recovery rates than those admitted in stabilisation centres. The recovery rate did vary by type of feeding programs for children with severe wasting.

## 4. Discussion

The study sought to document good practices and generate evidence on the effectiveness and scalability of CMSW programs in South Sudan. We found evidence of best partnership practices [34], characterised by improved availability and expansion of CMSW programs; the development of national protocols and standardisation of CMSW program implementation approaches; improved competence through training opportunities for primary caregivers, government and NGO partner staff, and health workers on IYCF, and the provision of multi-agency technical assistance and coordination. Adopting a broader national strategy to CMSW program implementation facilitated scale-up and expansion, and maximised collaboration, reach and quality of the response. These findings suggest that the implementation of CMSW programs in South Sudan has been consistent with best practice and the Humanitarian Charter and Minimum Standards in Humanitarian Response [13].

Nonetheless, the study found that there were barriers to strengthening the health systems, engaging communities and government structures, and funding and financial sustainability. One of the most documented challenge to implementing CMSW programs in resource-constrained countries is weak community mobilisation [35]. Using the healthcare access and quality (HAQ) index measured on a scale from 0 (worst) to 100 (best) as a component of achieving universal health coverage, South Sudan has consistently recorded a meagre HAQ index, estimated at just 26.8, which is among the lowest over time [36]. The country did not record significant gains during the millennium development goals era and is predicted to fall behind in the sustainable development goals era [36]. With a shrinking fiscal policy space and inadequate health spending, health governance and service models are compromised, often compounded by a fragmented health system that depends heavily on UN agencies, international NGOs, and other humanitarian aid organisations [37]. These challenges make it difficult to engage communities and government departments as co-designers and co-implementers and to embed CMSW programs fully into national health systems.

Our findings suggest that, despite the ongoing unrest and insurgency, the emergency response has been under control. However, the prevalence of wasting remained high due to a combination of many factors. The latest FSNMS survey [12] found that, at the individual level, wasting was not associated with food intake but was significantly lower among children who were vaccinated against measles and received deworming tablets than those who did not. However, high levels of wasting were significantly associated with child illness (diarrhoea, cough, fever and other infections). At the household level, high levels of wasting were associated with very high food expenditures, lack of latrines and practicing open defecation, female-headed households, and households with returnees. To maximise and sustain the impact of nutrition emergency preparedness and response programs in South Sudan will require significant investments in integrated water, sanitation and hygiene infrastructure, and behaviour change programs.

Exit outcomes for CMSW programs in South Sudan far exceed SPHERE minimum standards and compare favourably with results from other African regions [22,23,38,39]. Although case fatality rates among children with severe wasting globally have historically remained unacceptably high [35,40], in our study, the reported exit outcome estimates were a high recovery rate of 86.4%, a low case fatality rate of 2.1%, a low defaulter rate of 5.2%, and a low relapse rate of 2.2%. Where high recovery and low relapse rates are observed, it is expected that low mortality and low defaulter rates will be achieved [38], which is consistent with our findings. High case fatality and relapse rates in CMSW programs are often closely associated with limited access to medical care, including inappropriate or early discharge practices, unavailability of essential drugs, and lower parental awareness of nutrition-related medical complications requiring urgent and timely treatment [41]. Therefore, our study’s high recovery and low case fatality and relapse rates reflect well-resourced emergency programs. This success was a result of many interlinked activities. These encompassed early identification and referral of children with severe wasting before developing complications, appropriate management of admitted children including routine and systematic treatment as well as adherence to therapeutic feeding protocols. These efforts were supported by an integrated and harmonised multi-agency and multidisciplinary approach to service delivery, and supportive technical assistance. The low defaulter rate indicates an effective tracing of beneficiaries and follow-up, and accessibility of CMSW programs.

Our study found a low rate for non-recovery (1.7%) and medical transfers (4.6%), which could indicate better management of chronic infectious diseases. On admission, children with severe wasting usually receive a systematic treatment that encompasses the treatment of bacterial infections with broad-spectrum antibiotics, de-worming, malaria prevention in endemic areas, correction of electrolyte disturbances (e.g., ReSoMal-Rehydration Solution for Malnutrition) and micronutrient deficiencies (e.g., vitamin A supplementation and antianemia medications such as folic acid and iron), and measles immunisation. However, various studies have reported the increased severity of chronic infectious diseases such as tuberculosis and HIV/AIDS in children with severe wasting [35,42,43]. The diagnosis and management of chronic infectious diseases are not part of the systematic treatment. Their diagnosis and management operate independently of CMSW programs, which may delay case identification and timely referral for treatment, leading to slow recovery [35]. Our results of low non-recovery and medical transfer rates could indicate better management of comorbid chronic infectious disease, better coordination and integration of health and nutrition intervention systems, effective linkages with and referrals to external specialised care for chronic infectious disease. It could also indicate good monitoring and management of food sharing at the household level.

The weight gain velocity of 3.3 g/kg/day was well below the prescribed SPHERE minimum standards (i.e., ≥8 g/kg/day), which is consistent with findings summarised in systematic reviews [20,21,44]. It is interesting to note that lower weight gain velocity has generally been reported in CMSW programs across the board. Yebyo and colleagues [23] reported an overall weight gain velocity of 5.2 g/kg/day for OTPs in Ethiopia. In Nigeria, Chitekwe et al. [22] reported a weight gain velocity of 3.6 g/kg/day. In Collins’s review [38] of key issues in the success of community-based management of severe wasting involving 30 studies, none of the included studies met SPHERE minimum standards for weight gain velocity. Similarly, Ashworth [21] examined the efficacy and effectiveness of CMSW programs using data from 33 studies on community-based rehabilitation. Only six programs achieved an average weight gain velocity of more than 5 g/kg/day and were mainly experimental studies (randomised controlled trials). Inadequate weight gain velocity has also been reported in Asian countries such as Bangladesh (3.2 g/kg/day) [45] and India (5.1 g/kg/day) [46].

This pattern of weight gain velocity is more associated with the effectiveness of monitoring and evaluation systems in tracking admission and discharge criteria rather than the performance of CMSW programs per se. For example, adequate weight gain velocity was observed in programs that provided a domiciliary ration of RUTF sufficient to meet the needs for catch-up growth (i.e., 175 kcal/kg/day or 1000 kcal/day) [21] and consistently used the same indicator for admissions and discharges [44,45]. These programs used WHZ or MUAC as a stand-alone criterion for admitting and discharging children. In the case of South Sudan, children were often admitted into CMSW programs using two parallel admission and discharge criteria. That is, within the same centres, children could be admitted and discharged based either on WHZ, or mid-upper arm circumference (MUAC). Some children were admitted on MUAC and monitored using MUAC until discharge; whilst others were admitted on WHZ and followed up during treatment using the same index until discharged. The use of two different anthropometric indices within the same centres made the standardisation of the weight gain velocity a challenge. Similarly, the improved reporting system in South Sudan only captured site-based aggregated data. Aggregated data were not distinguished by admission and discharge criteria, and came from multiple centres by differing implementing NGO partners, which distorted the accuracy of the weight gain velocity in the absence of individual-level data. Therefore, the evidence of weight gain velocity as an effectiveness indicator in CMSW programs remains insufficient and somehow controversial.

The reported length of stay in our programs is within the standards of the South Sudanese Government’s protocol for managing severe wasting. The government’s protocols set the length of stay for children with severe wasting admitted in CMSW to 6–8 weeks [31]. However, the government allowed an additional one month to account for the fragile environment in which CMSW programs were implemented. This decision had as an impact on length of stay as an indicator. Factors that influenced this decision included high levels of food insecurity, a high likelihood of families selling and sharing of RUTF, and absenteeism for scheduled follow-up visits (mainly due to insecurity and displacement) [31]. The length of stay in CMSW programs in our study averaged 6.7 weeks, which met the government’s minimum standards. It was comparable to figures reported in other African regions (6–7 weeks) [22,23,38,44], but lower than those reported in Pakistan (10 weeks) [45] and India (7.3–8.7 weeks) [46].

The study found that our results on exit outcome indicators did not vary by sex and age. However, children whose sex were categorised as “other” were less likely to recover and more likely to die during treatment or transferred for medical treatment than girls. They were children more likely to be aged 5 years or older, hence falling outside the target age group for CMSW programs in South Sudan. Whilst most CMSW programs admit children aged 5 years or older, there are still insufficient guidelines related to admission and discharge criteria as well as therapeutic feeding protocol. Nevertheless, the South Sudanese CMSW guidelines provide technical notes on this issue [31]. The guidelines state that older children admitted to CMSW should be treated using the same basic protocols as inpatient and OTPs, including routine and systematic treatment on admission and therapeutic foods according to their weight. Admission criteria are based on MUAC (<135 mm for 5–9 years or <160 mm if 10–14 years), or body mass index [BMI] for age (BMI-for-age < −3 z-scores), or the presence of bilateral pitting oedema. The same criteria are used to guide discharge protocols, that is, BMI-for-age ≥ −3 z-scores for 2 consecutive visits, MUAC ≥ 135 mm if 5–9 years and ≥ 160 mm if 10–14 years for 2 consecutive visits, or no bilateral pitting oedema for 2 consecutive visits and the child is clinically well and alert. Despite that such guidelines exist, the misclassification of the demographic profile of children aged 5 years or older is concerning. Our findings suggest that poor classification of demographic factors could contribute to health inequities. CMSW in South Sudan will need to be people-centred and gender-sensitive to address challenges that marginalised groups or those considered outside of mainstream society may face.

CMSW program performance indicators improved significantly over time, suggesting that key actors have effectively integrated lessons learned into policies, practice, and strategic plans. Nonetheless, regional differences were evident. Northern Bahr el Ghazal performed poorly. It had a high relapse rate and lower recovery rate. The relapse rate can be explained by poor linkage and referral between OTP and supplementary feeding programs as well as sub optimal care practices at home. The poor recovery rate observed in this state could be due to underlying morbidities and sharing of therapeutic foods with other family members. The states of Lakes, Jonglei, Unity, and Warrap had lower case fatality rates than Central Equatoria, which could be due to increased coverage and screening for early case identification and referral for treatment. Eastern Equatoria, Lakes, Jonglei, and Western Bahr el Ghazal had lower medical transfer rates. However, these states are very remote so lower medical transfer rates could do simply due to under-documentation, hence under-representation of wasting comorbidities. Children admitted in OTPs recorded lower deaths and medical transfer rates but higher default and non-recovery rates than those admitted in stabilisation centres. This finding could be linked to admission criteria. OTP sites admitted children with severe wasting without medical complications while stabilisation centres admitted children with medical complications, hence had an increased risk of mortality.

The study has some limitations as well as strengths. The biggest strength is the longitudinal nature of our data, with large sample sizes. Some of the weaknesses are inherent in the implementation of CMSW programs. Data collection for monitoring and evaluation purposes varied across programs. Due to the multitude of implementing NGO partners with differing organisational missions, funding schemes, and capacity, data on therapeutic feeding and treatment practices (e.g., RUTF or routine medications), treatment compliance at the home-level (e.g., food sharing), and clinical features were not routinely collected. Where they existed, they were unreliable. This made it difficult to assess how these factors were associated with exit outcome indicators. Data used for the analyses in this study were based on aggregated secondary data derived from a combination of individual-level data from a multitude of NGOs involved in implementing CMSW programs. The quality of data varied significantly and limited our ability to undertake analyses at the individual level. For example, whilst the FSNMS survey methodology evolved over the years, the sampling approaches and the quality of data were inconsistent within and between rounds and states. The SMART surveys were conducted annually at county level and could not be representative of states. They were carried out annually in priority target counties, but irregularly implemented in the least affected counties. While SMART surveys provided a snapshot of the CMSW programs’ impact on indicators sensitive to changes in the overall health status such as mortality, our mortality data need to be interpreted with caution. Finally, there are currently no thresholds to guide the interpretation of relapse, medical transfers, and non-recovery rates, limiting our understanding of the impact of these indicators on revising targeting approaches and/or refocusing treatment. Notwithstanding these limitations, our results compare favourably with those reported in the literature [38]. The experience in South Sudan demonstrates that the evaluation of CMSW programs should not merely focus on exit outcomes but must include the documentation of best practice and encompass an integrated multi-dimensional approach to include indicators of nutrition emergency preparedness and severe wasting prevention, treatment, and management.

## 5. Conclusions

There was better coordination of CMSW program implementation, which facilitated the provision of timely and quality care through an integrated and harmonised multi-agency and multidisciplinary approach. Our findings suggest the possibility of implementing resilient CMSW programs in protracted conflict environments, informed by global guidelines and protocols. Although national guidelines and protocols exist in South Sudan, the weak health system, fragile health budget that relies on external assistance, and limited opportunities for competency-based learning and knowledge transfer makes the institutionalisation and ownership of CMSW programs difficult. To achieve this objective may require significant paradigm shifts to develop customised sustainable and effective system-strengthening efforts and models of CMSW program implementation that reflect the socio-political environment and capacity of target areas.

## Figures and Tables

**Table 1:** Enablers of stabilisation centres and OTPs’ scalability.

Scaling up Process	Operational Documents	Implementation Plans/Reports	Strategic Plan	Monitoring and Evaluation Plans and Documents	National Guidelines	Field and Assessment Reports
Intervention delivery pathways and harmonisation of implementation plans	×	√	×	√	√	√
Delivery system: reach, adoption, and expansion	√	√	√	√	√	√
Providing technical assistance and strong organisational capacity at all levels	√	√	√	√	√	×
Integrating approaches within government systems, policies, priorities, and targets	×	√	×	×	√	×
Generating and disseminating evidence (effectiveness)	×	√	×	√	×	√
Engaging communities as co-designers and co-implementers (ownership)	×	×	×	×	×	×
Using monitoring and evaluation systems to inform practice and policy	√	√	√	√	√	√
Building an enabling environment and strengthening the decision support	√	√	√	√	√	√
Facilitating partnerships and integration	√	√	√	√	√	√
Delineation of roles and responsibilities	√	√	√	√	√	√
Funding and financial sustainability	×	×	×	×	×	×

√ = evident and mentioned in detail, × = inexistent and not mentioned.

**Table 2:** Inventory of severe wasting prevention and nutrition emergency preparedness activities.

Domain	Indicator	2014	2015	2016	2017	2018	2019	2020
**Infant and young child feeding (IYCF) practices (*N*)**								
	Target: IYCF counselling for primary caregivers of children aged 0–23 months	No target	230,698	567,366	590,134	1,201,386	984,701	1,098,241
	Trained: IYCF counselling for primary caregivers of children aged 0–23 months	118,216 (N/A)	539,547 (233.9%)	724,500 (127.7%)	607,539 (102.9%)	950,376 (79.1%)	1,684,197 (171.0%)	1,644,323 (149.7%)
	# of Government and NGO partner staff and health workers trained in IYCF	No data	3000	2990	1885	2831	4189	3408
	% counties that have carried out vitamin A supplementation (79 counties)	43%	51%	43%	61%	91%	98.7%	84.8%
**Nutrition Emergency Preparedness and Response (*N*)**								
	Outpatient therapeutic program sites established	351	462	580	736	858	1145	1171
	UNICEF IRRM missions	37	59	34	66	50	32	11
	Children aged 6–59 months screened for wasting during IRRM missions	92,715	131,545	84,099	114,674	76,550	65,869	11,110
	Wasted children treated during IRRM missions *	2886 (3.1%)	1275 (1.0%)	382 (0.5%)	1797 (1.6%)	1110 (1.5%)	557 0.8%)	367 (3.3%)
	Caregivers receiving maternal and child nutrition education during IRRM missions	20,478	35,804	19,992	55,052	35,350	11,640	10,332
	Children aged 6–59 months supplemented with Vitamin A during IRRM missions	47,057 (50.8%)	74,808 (56.9%)	24,772 (29.5%)	52,999 (46.2%)	45,178 (59.0%)	42,479 (65.5%)	5543 (49.9%)
	12–59 month-old children who received deworming tablets during IRRM missions	40,355 (43.5%)	62,278 (47.3%)	27,861 (33.1%)	44,301 (38.6%)	34,211 (44.7%)	34,369 (52.2%)	6204 (55.8%)
**Evidence based nutrition intervention:**								
	Average reporting rate of outpatient therapeutic program (%)	NA	NA	86%	90%	95%	95%	95%
	SMART surveys validated by UNICEF/Nutrition Information Working Group	42 (100%)	59 (100%)	56 (100%)	55 (100%)	55 (100%)	28 (100%)	5 (100%)
	Existence of a national costed nutrition strategic plan	No	No	No	No	No	RD **	RD **
	Existence of a national management information system	Yes	Yes	Yes	Yes	Yes	Yes	

* Exclude cases of children with severe wasting in CMSW implemented by NGO implementing partners; ** RD = Roadmap Developed.

**Table 3:** Performance indicators of CMSW.

Indicators	2016	2017	2018	2019	2020	All Years	Minimum Standards *
**Sex**							
**Girls**							
Total admissions, *N*	112,487	113,962	110,751	129,873	109,741	576,814	
Total exits, *N*	92,241 (82.00)	90,487 (79.40)	93,322 (84.26)	111,416 (85.79)	91,935 (83.77)	479,401 (83.11)	
Relapse rate [mean (SD] **	1.30 (2.28)	2.31 (5.67)	1.85 (3.30)	2.48 (3.59)	2.22 (3.35)	2.06 (3.83)	<10%
**Exits [mean (SD]**							
Recovered	83.93 (18.55)	83.89 (18.21)	86.95 (17.73)	89.38 (14.10)	90.94 (15.06)	87.22 (16.92)	>75%
Died	1.16 (3.50)	1.86 (5.72)	2.32 (9.32)	1.96 (8.50)	1.61 (5.21)	1.81 (6.92)	<10%
Defaulters	7.48 (11.83)	6.91 (12.77)	4.58 (7.20)	3.87 (5.77)	2.19 (3.27)	4.85 (8.87)	<15%
Not- recovered	1.95 (3.50)	1.8 (5.01)	2 (7.93)	1.47 (2.44)	1 (2.06)	1.63 (4.76)	
Transferred	5.49 (13.04)	5.54 (12.8)	4.15 (11.02)	3.33 (10.17)	4.26 (14.37)	4.50 (12.34)	
Weight gain velocity	3.51 (2.91)	1.09 (2.20)	2.86 (5.02)	4.17 (4.11)	3.30 (2.68)	3.01 (3.72)	≥8 g/kg/day
LOS in the program ***	4.81 (2.48)	6.68 (4.89)	5.11 (2.75)	4.81 (2.59)	5.12 (2.79)	5.29 (3.27)	<8 weeks
**Boys**							
Total admissions, *N*	103,833	103,114	102,458	118,909	95,370	523,684	
Total exits, *N*	83,029 (79.96)	83,486 (80.96)	86,181 (84.11)	104,046 (87.50)	80,853 (84.78)	437,595 (83.56)	
Relapse rate [mean (SD]	1.21 (2.10)	2.30 (4.87)	2.48 (5.28)	2.98 (5.17)	2.44 (4.57)	2.33 (4.65)	<10%
**Exits [mean (SD]**							
Recovered	84.94 (16.77)	83.92 (18.49)	87.59 (13.64)	88.07 (14.65)	91.71 (13.29)	87.43 (15.56)	>75%
Died	2.46 (9.69)	2.3 (8.70)	1.54 (4.06)	1.87 (4.56)	1.72 (4.85)	1.95 (6.57)	<10%
Defaulters	6.79 (10.74)	6.93 (10.18)	5.51 (9.86)	4.13 (6.07)	2.26 (3.21)	5.01 (8.54)	<15%
Not- recovered	1.91 (3.76)	1.7 (4.41)	1.44 (2.60)	1.53 (4.00)	0.82 (1.55)	1.45 (3.39)	
Transferred	3.91 (8.84)	5.14 (13.38)	3.92 (9.28)	4.41 (12.94)	3.49 (12.08)	4.16 (11.50)	
Weight gain velocity	3.57 (3.00)	1.27 (2.66)	2.94 (4.27)	3.93 (3.03)	3.41 (2.80)	3.04 (3.35)	≥8 g/kg/day
LOS in the program	4.72 (2.41)	6.87 (5.43)	5.17 (2.73)	4.76 (2.50)	5.09 (2.71)	5.31 (3.41)	<8 weeks
**Other**							
Total admissions, *N*	1787	784	1256	572	649	5048	
Total exits, *N*	356 (19.92)	663 (84.57)	639 (50.88)	570 (99.65)	514 (79.20)	2751 (54.50)	
Relapse rate [mean (SD]	1.42 (8.33)	2.31 (5.50)	1.38 (4.47)	2.02 (7.67)	3.35 (9.15)	2.09 (7.21)	<10%
**Exits [mean (SD]**							
Recovered	79.88 (28.94)	76.15 (29.17)	74.83 (37.42)	83.82 (28.73)	82.72 (33.26)	79.56 (32.12)	>75%
Died	4.84 (13.66)	1.58 (5.70)	5.56 (17.86)	2.05 (6.47)	3.3 (16.02)	3.57 (13.29)	<10%
Defaulters	9.26 (25.60)	8.16 (11.73)	6.02 (15.57)	5.01 (15.68)	7.62 (23.46)	6.87 (18.60)	<15%
Not- recovered	3.16 (8.02)	4.57 (9.84)	1.95 (8.14)	5.14 (19.95)	1.43 (7.96)	3.20 (12.33)	
Transferred	2.86 (10.60)	9.54 (25.95)	11.64 (30.15)	3.98 (16.23)	4.93 (17.78)	6.30 (21.96)	
Weight gain velocity	3.53 (2.83)	1.15 (2.01)	2.03 (11.66)	4.07 (3.77)	3.65 (5.08)	2.90 (6.32)	≥8 g/kg/day
LOS in the program	4.87 (2.47)	6.63 (5.24)	5.15 (2.90)	4.94 (2.59)	5.11 (2.50)	5.32 (3.34)	<8 weeks
**Age group**							
**<6 months**							
Total admissions, *N*	443	440	735	787	524	2929	
Total exits, *N*	352 (79.46)	438 (99.55)	730 (99.32)	455 (57.81)	458 (87.40)	2433 (83.07)	
Relapse rate [mean (SD]	0.21 (1.14)	3.44 (10.41)	2.34 (7.54)	2.21 (5.13)	1.06 (3.11)	1.91 (6.64)	<10%
**Exits [mean (SD]**							
Recovered	82.24 (31.43)	82.21 (26.80)	89.02 (22.73)	85.13 (30.04)	90.42 (21.3)	86.05 (26.23)	>75%
Died	4.13 (15.7)	3.17 (8.43)	3.39 (13.85)	3.99 (16.41)	2.87 (9.57)	3.46 (12.80)	<10%
Defaulters	7.89 (21.98)	4.03 (15.07)	1.36 (3.73)	0.93 (3.38)	0.69 (2.80)	2.86 (11.97)	<15%
Not- recovered	0 (0)	0.15 (1.15)	1.92 (12.09)	0.1 (0.62)	0.17 (1.23)	0.58 (6.26)	
Transferred	5.73 (17.72)	10.44 (23.35)	4.3 (12.61)	9.86 (26.79)	5.85 (19.71)	7.05 (20.01)	
Weight gain velocity	3.41 (2.57)	1.16 (2.21)	2.77 (5.73)	4.03 (3.59)	3.20 (2.54)	2.91 (3.76)	≥8 g/kg/day
LOS in the program	4.90 (2.45)	6.81 (5.24)	5.12 (2.67)	4.98 (2.86)	5.15 (2.88)	5.39 (3.43)	<8 weeks
**6–59 months**							
Total admissions, *N*	215,877	216,636	212,474	247,995	204,587	1,097,569	
Total exits, *N*	174,918 (81.03)	173,520 (80.10)	178,728 (84.12)	215,007 (86.70)	172,330 (84.23)	914,503 (83.32)	
Relapse rate [mean (SD]	1.51 (2.31)	2.03 (2.91)	2.12 (3.03	2.83 (4.34)	2.57 (4.11)	2.25 (3.52)	<10%
**Exits [mean (SD]**							
Recovered	84.89 (13.1)	84.34 (15.53)	86.8 (13.39)	89.25 (10.31)	91.5 (12.44)	87.59 (13.23)	>75%
Died	1.31 (3.36)	1.81 (7.05)	1.54 (3.78)	1.61 (3.79)	1.44 (3.57)	1.57 (4.48)	<10%
Defaulters	6.98 (7.33)	7.65 (10.35)	6.02 (9.28)	4.44 (6.07)	2.51 (3.23)	5.37 (7.78)	<15%
Not- recovered	2.34 (3.87)	2.15 (5.16)	1.67 (2.36)	1.7 (3.48)	1.05 (1.88)	1.74 (3.500	
Transferred	4.49 (9.26)	4.06 (8.41)	3.97 (9.45)	3 (6.85)	3.5 (11.67)	3.76 (9.29)	
Weight gain velocity	3.60 (3.13)	1.20 (2.54)	2.97 (4.02)	4.06 (3.63)	3.43 (2.82)	3.07 (3.43)	≥8 g/kg/day
LOS in the program	4.70 (2.44)	6.76 (5.12)	5.15 (2.77)	4.69 (2.38)	5.09 (2.69)	5.26 (3.30)	<8 weeks
**>=60 months**							
Total admissions, *N*	1787	784	1256	572	649	5048	
Total exits, *N*	356 (19.92)	663 (84.57)	639 (50.88)	572 (100.00)	514 (79.20)	2744 (54.36)	
Relapse rate [mean (SD]	1.42 (8.33)	2.31 (5.50)	1.38 (4.47)	2.02 (7.67)	3.35 (9.15)	2.09 (7.21)	<10%
**Exits [mean (SD]**							
Recovered	79.88 (28.94)	76.15 (29.17)	74.83 (37.42)	83.82 (28.73)	82.72 (33.26)	79.56 (32.12)	>75%
Died	4.84 (13.66)	1.58 (5.70)	5.56 (17.86)	2.05 (6.47)	3.3 (16.02)	3.57 (13.12)	<10%
Defaulters	9.26 (25.6)	8.16 (11.73)	6.02 (15.57)	5.01 (15.68)	7.62 (23.46)	6.87 (18.60)	<15%
Not- recovered	3.16 (8.02)	4.57 (9.84)	1.95 (8.14)	5.14 (19.95)	1.43 (7.96)	3.20 (12.33)	
Transferred	2.86 (10.6)	9.54 (25.95)	11.64 (30.15)	3.98 (16.23)	4.93 (17.78)	6.80 (21.96)	
Weight gain velocity	3.60 (2.89)	1.40 (2.34)	1.19 (2.03)	4.12 (3.80)	3.50 (4.98)	3.01 (5.87)	≥8 g/kg/day
LOS in the program	4.99 (2.59)	6.68 (5.30)	6.70 (5.31)	4.98 (2.60)	5.18 (2.57)	5.40 (3.42)	<8 weeks
**All**							
Total admissions, *N*	218,107	217,860	214,465	249,354	205,760	1,105,546	
Total exits, *N*	175,626 (80.52)	174,636 (80.16)	180,142 (84.00)	216,041 (86.64)	173,302 (84.23)	919,747 (83.19)	
Relapse rate [mean (SD]	1.27 (3.46)	2.31 (5.29)	2.06 (4.43)	2.65 (4.95)	2.45 (4.91)	2.18 (4.70)	<10%
**Exits [mean (SD]**							
Recovered	83.96 (19.12)	83.24 (19.55)	85.59 (20.48)	88.07 (17.02)	90.44 (17.3)	86.44 (18.89)	>75%
Died	2.11 (8.18)	2.04 (7.22)	2.42 (9.41)	1.93 (6.77)	1.83 (6.99)	2.07 (7.78)	<10%
Defaulters	7.35 (13.43)	7.03 (11.53)	5.17 (9.83)	4.13 (7.91)	2.78 (8.24)	5.15 (10.32)	<15%
Not- recovered	2.06 (4.29)	1.99 (5.38)	1.75 (6.24)	1.98 (7.94)	0.96 (3.07)	1.73 (5.71)	
Transferred	4.51 (11.11)	5.7 (14.62)	5.06 (14.72)	3.88 (12.31)	3.98 (13.78)	4.61 (13.46)	
Weight gain velocity	3.47 (23.73)	3.08 (3.29)	2.81 (4.29)	3.34 (3.62)	3.43 (3.49)	3.30 (14.97)	≥8 g/kg/day
LOS in the program	6.91 (3.87)	6.61 (3.26)	5.88 (2.65)	5.67 (2.76)	5.12 (2.67)	6.65 (3.65)	<8 weeks

* SPHERE Minimum Standards in Humanitarian Response. ** LOS= Length of stay; Standards based on the South Sudan protocol for management of severe wasting, which stipulates children with severe wasting to stay in the OTP for a maximum of eight weeks. *** SPHERE standards do not stipulate minimum standards for relapse after recovery. The 10% is extracted from the literature.

**Table 4:** Unadjusted models exploring factors associated with exit outcome indicators and relapse.

Variable	Recovered				Died				Defaulted				Non-Recovered				Transferred				Readmitted			
	β	95% CI		*p*-Value	β	95% CI		*p*-Value	β	95% CI		*p*-Value	β	95% CI		*p*-Value	β	95% CI		*p*-Value	β	95% CI		*p*-Value
Sex																								
Girls	Ref				Ref				Ref				Ref				Ref				Ref			
Boys	0.21	−1.62	2.05	0.820	0.14	−0.62	0.90	0.720	0.16	−0.85	1.17	0.762	−0.18	−0.73	0.38	0.536	−0.33	−1.65	0.98	0.620	0.27	−0.18	0.73	0.239
Other	−7.66	−10.53	−4.78	0.000	1.77	0.58	2.96	0.004	2.02	0.44	3.60	0.012	1.57	0.70	2.44	0.000	2.30	0.24	4.36	0.029	0.04	−0.67	0.74	0.915
**Age-group**																								
<6 months	Ref				Ref				Ref				Ref				Ref				Ref			
6–59 months	1.53	−0.88	3.95	0.214	−1.91	−2.91	−0.91	0.000	2.51	1.18	3.84	0.000	1.16	0.43	1.89	0.002	−3.29	−5.02	−1.56	0.000	0.34	−0.25	0.93	0.256
>=60 months	−6.50	−9.87	−3.12	0.000	0.12	−1.28	1.51	0.869	4.01	2.16	5.87	0.000	2.62	1.59	3.64	0.000	−0.25	−2.67	2.16	0.839	0.18	−0.64	1.00	0.667
**Years**																								
2016	Ref				Ref				Ref				Ref				Ref				Ref			
2017	−0.72	−3.63	2.19	0.629	−0.07	−1.29	1.14	0.904	−0.33	−1.91	1.26	0.685	−0.07	−0.95	0.82	0.885	1.18	−0.91	3.28	0.267	1.04	0.32	1.75	0.004
2018	1.63	−1.17	4.43	0.253	0.30	−0.86	1.47	0.610	−2.18	−3.71	−0.66	0.005	−0.30	−1.15	0.55	0.487	0.55	−1.47	2.56	0.595	0.79	0.11	1.48	0.024
2019	4.11	1.28	6.94	0.004	−0.18	−1.36	0.99	0.759	−3.22	−4.76	−1.68	0.000	−0.07	−0.94	0.79	0.865	−0.63	−2.67	1.40	0.542	1.38	0.68	2.08	0.000
2020	6.48	3.65	9.30	0.000	−0.28	−1.45	0.90	0.641	−4.57	−6.11	−3.03	0.000	−1.09	−1.95	−0.23	0.013	−0.53	−2.56	1.50	0.607	1.18	0.49	1.88	0.001
**States**																								
Central Equatoria	Ref				Ref				Ref				Ref				Ref				Ref			
Eastern Equatoria	7.52	3.38	11.66	0.000	−0.18	−1.92	1.57	0.844	−3.91	−6.22	−1.61	0.001	−1.18	−2.47	0.10	0.071	−2.25	−5.20	0.70	0.136	0.35	−0.67	1.38	0.500
Lakes	12.93	8.76	17.10	0.000	−1.84	−3.60	−0.07	0.041	−5.25	−7.57	−2.93	0.000	−1.39	−2.68	−0.09	0.035	−4.45	−7.43	−1.48	0.003	−0.51	−1.55	0.54	0.343
Jonglei	13.76	9.86	17.66	0.000	−2.95	−4.60	−1.30	0.000	−6.23	−8.40	−4.06	0.000	−0.63	−1.84	0.58	0.309	−3.94	−6.73	−1.16	0.005	0.05	−0.91	1.02	0.912
Northern Bahr el Ghazal	−1.61	−6.14	2.93	0.487	−0.82	−2.74	1.09	0.401	−6.43	−8.95	−3.91	0.000	0.28	−1.13	1.69	0.697	8.58	5.34	11.81	0.000	2.50	1.37	3.64	0.000
Unity	12.76	8.72	16.80	0.000	−2.13	−3.84	−0.43	0.014	−6.95	−9.19	−4.70	0.000	−1.45	−2.70	−0.19	0.024	−2.23	−5.11	0.65	0.129	−0.20	−1.21	0.81	0.699
Upper Nile	9.23	5.11	13.36	0.000	−1.76	−3.51	−0.02	0.047	−4.01	−6.31	−1.72	0.001	−0.89	−2.17	0.40	0.176	−2.57	−5.52	0.37	0.086	−0.58	−1.59	0.44	0.266
Warrap	6.50	2.35	10.65	0.002	−1.64	−3.39	0.11	0.067	−4.57	−6.88	−2.27	0.000	−1.06	−2.35	0.22	0.106	0.78	−2.18	3.74	0.606	1.03	−0.01	2.06	0.053
Western Bahr el Ghazal	10.40	4.78	16.02	0.000	−1.81	−4.18	0.56	0.135	−3.44	−6.56	−0.31	0.031	0.12	−1.62	1.87	0.892	−5.28	−9.28	−1.27	0.010	0.31	−1.10	1.72	0.668
Western Equatoria	9.81	5.60	14.03	0.000	−0.90	−2.68	0.88	0.321	−5.89	−8.24	−3.55	0.000	−0.50	−1.81	0.81	0.456	−2.52	−5.53	0.49	0.101	−0.46	−1.52	0.59	0.392
**Type of program**																								
SC	Ref				Ref				Ref				Ref				Ref				Ref			
OTP	0.23	−1.52	1.97	0.799	−2.90	−3.61	−2.20	0.000	4.67	3.74	5.60	0.000	2.22	1.70	2.74	0.000	−4.21	−5.44	−2.98	0.000	0.86	0.43	1.28	0.000
**Length of stay**																								
<=5 weeks	Ref				Ref				Ref				Ref				Ref				Ref			
6–8 Weeks	−1.21	−4.09	1.68	0.412	0.28	−0.91	1.47	0.648	0.22	−1.36	1.80	0.784	0.15	−0.72	1.02	0.739	0.56	−1.50	2.62	0.593	0.32	−0.38	1.03	0.367
>=9 weeks	2.06	−0.31	4.43	0.088	−0.27	−1.25	0.71	0.588	−0.37	−1.66	0.93	0.578	−0.42	−1.13	0.30	0.254	−1.01	−2.70	0.68	0.242	0.22	−0.36	0.81	0.456
**Weight velocity**																								
<=3 g/kg/day	Ref				Ref				Ref				Ref				Ref				Ref			
4–7 g/kg/day	−0.42	−2.41	1.58	0.680	−0.22	−1.05	0.60	0.593	−0.18	−1.27	0.91	0.743	−0.31	−0.92	0.29	0.306	1.14	−0.28	2.56	0.116	0.26	−0.23	0.75	0.298
>=8 g/gg/day	0.27	−3.07	3.61	0.875	0.19	−1.18	1.57	0.784	−0.01	−1.83	1.82	0.995	−0.48	−1.49	0.53	0.350	0.03	−2.36	2.41	0.983	0.22	−0.60	1.05	0.595

**Table 5:** Adjusted models exploring factors associated with exit outcome indicators and relapse.

Variable	Recovered				Died				Defaulted				Non-Recovered				Transferred				Readmitted			
	β	95% CI		*p*-Value	β	95% CI		*p*-Value	β	95% CI		*p*-Value	β	95% CI		*p*-Value	β	95% CI		*p*-Value	β	95% CI		*p*-Value
Sex																								
Girls	Ref				Ref				Ref				Ref				Ref				Ref			
Boys	0.21	−1.56	1.98	0.815	0.14	−0.60	0.89	0.703	0.14	−0.82	1.11	0.769	−0.19	−0.73	0.36	0.502	−0.31	−1.58	0.95	0.628	0.28	−0.17	0.73	0.219
Other	−7.01	−10.66	−3.35	0.000	2.27	0.73	3.80	0.004	0.93	−1.06	2.92	0.361	0.94	−0.19	2.07	0.103	2.87	0.26	5.49	0.031	−0.50	−1.42	0.41	0.282
**Age-group**																								
<6 months	Ref				Ref				Ref				Ref				Ref				Ref			
6–59 months	0.61	−2.00	3.22	0.645	−0.10	−1.19	1.00	0.863	−0.04	−1.46	1.39	0.961	−0.10	−0.91	0.71	0.811	−0.38	−2.25	1.48	0.687	−0.24	−0.89	0.40	0.462
>=60 months	NED				NED				NED				NED				NED				NED			
**Years**																								
2016	Ref				Ref				Ref				Ref				Ref				Ref			
2017	−1.25	−4.15	1.65	0.399	−0.24	−1.46	0.98	0.703	−0.12	−1.70	1.46	0.881	0.02	−0.88	0.91	0.972	1.59	−0.48	3.66	0.133	1.04	0.31	1.77	0.005
2018	1.73	−0.99	4.45	0.213	−0.04	−1.19	1.11	0.945	−2.11	−3.59	−0.62	0.005	−0.24	−1.08	0.60	0.572	0.66	−1.29	2.61	0.506	0.96	0.27	1.64	0.006
2019	4.20	1.45	6.96	0.003	−0.38	−1.54	0.78	0.522	−3.18	−4.68	−1.67	0.000	0.01	−0.84	0.87	0.973	−0.66	−2.63	1.31	0.509	1.48	0.79	2.17	0.000
2020	6.34	3.60	9.08	0.000	−0.53	−1.68	0.62	0.367	−4.41	−5.90	−2.91	0.000	−0.98	−1.83	−0.13	0.024	−0.43	−2.39	1.53	0.669	1.33	0.65	2.02	0.000
**States**																								
Central Equatoria	Ref				Ref				Ref				Ref				Ref				Ref			
Eastern Equatoria	7.14	3.04	11.25	0.001	−0.41	−2.14	1.32	0.640	−3.59	−5.83	−1.35	0.002	−0.99	−2.26	0.28	0.126	−2.15	−5.09	0.78	0.151	0.46	−0.56	1.48	0.378
Lakes	12.54	8.42	16.66	0.000	−2.03	−3.77	−0.30	0.021	−4.88	−7.13	−2.64	0.000	−1.13	−2.40	0.15	0.083	−4.50	−7.44	−1.56	0.003	−0.33	−1.37	0.71	0.537
Jonglei	13.00	9.12	16.87	0.000	−3.06	−4.69	−1.43	0.000	−5.87	−7.98	−3.75	0.000	−0.36	−1.56	0.84	0.555	−3.71	−6.48	−0.94	0.009	0.20	−0.77	1.16	0.689
Northern Bahr el Ghazal	−1.52	−6.03	2.98	0.507	−1.20	−3.09	0.70	0.215	−6.25	−8.71	−3.80	0.000	0.47	−0.92	1.86	0.509	8.51	5.29	11.73	0.000	2.77	1.63	3.91	0.000
Unity	12.37	8.37	16.37	0.000	−2.42	−4.10	−0.74	0.005	−6.46	−8.64	−4.28	0.000	−1.20	−2.44	0.04	0.057	−2.29	−5.15	0.57	0.117	−0.03	−1.05	0.98	0.949
Upper Nile	8.25	4.14	12.36	0.000	−1.57	−3.30	0.16	0.075	−4.06	−6.30	−1.82	0.000	−0.82	−2.09	0.45	0.208	−1.80	−4.74	1.14	0.230	−0.42	−1.44	0.61	0.425
Warrap	6.30	2.18	10.43	0.003	−2.11	−3.85	−0.38	0.017	−3.68	−5.93	−1.43	0.001	−0.51	−1.79	0.76	0.432	0.00	−2.95	2.95	1.000	1.29	0.25	2.33	0.015
Western Bahr el Ghazal	10.72	5.17	16.28	0.000	−1.90	−4.23	0.44	0.112	−3.74	−6.77	−0.71	0.015	0.12	−1.60	1.84	0.891	−5.21	−9.18	−1.24	0.010	0.40	−1.00	1.81	0.576
Western Equatoria	8.86	4.65	13.08	0.000	−0.95	−2.73	0.82	0.291	−5.46	−7.75	−3.16	0.000	−0.39	−1.70	0.91	0.553	−2.06	−5.07	0.95	0.180	−0.45	−1.51	0.61	0.406
**Type of program**																								
SC	Ref				Ref				Ref				Ref				Ref				Ref			
OTP	0.90	−0.98	2.78	0.349	−3.17	−3.97	−2.38	0.000	4.40	3.38	5.43	0.000	2.10	1.52	2.68	0.000	−4.23	−5.58	−2.89	0.000	1.12	0.64	1.59	0.000
**Length of stay**																								
<=5 weeks	Ref				Ref				Ref				Ref				Ref				Ref			
6–8 Weeks	−1.06	−3.85	1.73	0.457	0.18	−1.00	1.35	0.765	0.19	−1.34	1.71	0.810	0.05	−0.81	0.92	0.904	0.64	−1.35	2.64	0.529	0.36	−0.34	1.06	0.310
>=9 weeks	2.10	−0.28	4.48	0.084	−0.35	−1.35	0.65	0.497	−0.44	−1.74	0.85	0.503	−0.48	−1.21	0.26	0.205	−0.84	−2.54	0.87	0.336	0.32	−0.28	0.92	0.293
**Weight velocity**																								
<=3 g/kg/day	Ref				Ref				Ref				Ref				Ref				Ref			
4–7 g/kg/day	−0.87	−2.93	1.18	0.406	−0.10	−0.96	0.77	0.825	−0.25	−1.37	0.87	0.667	−0.38	−1.01	0.26	0.246	1.59	0.12	3.06	0.034	0.18	−0.34	0.69	0.508
>=8 g/gg/day	−0.24	−3.53	3.04	0.886	0.23	−1.15	1.61	0.746	0.30	−1.49	2.09	0.741	−0.58	−1.60	0.44	0.263	0.29	−2.06	2.64	0.808	0.04	−0.79	0.88	0.919

NED = not enough data due to sample sizes.

## Data Availability

Restricted by UNICEF but available on request form monitoring and evaluation coordinator Qutab Alam qalam@unicef.org.

## Supplementary Materials

The following are available online at https://www.mdpi.com/article/10.3390/ijerph18179113/s1, Supplementary Materials S1: Reviewed documents.
