# Supplementary material for: Assessing the Impact of Integrated Community-Based Management of Severe Wasting Programs in Conflict-Stricken South Sudan: A Multi-Dimensional Approach to Scalability of Nutrition Emergency Response Programs
Source: Int J Environ Res Public Health. 2021 Aug 29;18(17):9113. doi: 10.3390/ijerph18179113 (PMC8431605; doi:10.3390/ijerph18179113)
Supplement: Supplementary file 1 [file ijerph-18-09113-s001.zip › ijerph-1310238-supplementary.pdf]

Appendix 1: Reviewed documents

1. Aburmishan D, Tyler V, Alamprese L, Nicholas P, Raphael M, Senesie J, Lawi A, Tesfaselassie K, Kassim I, Abebe K et al: Scaling-up of care for children with acute malnutrition during emergency nutrition response in South Sudan between 2014 and 2018. Field Exchange 2019, 59:73-77
2. Action Against Hunger. CMAM COVID Adaptations: South Sudan Case Study https://resources.acutemalnutrition.org/CMAM%20COVID%20Adaptations_South%20Sudan%20Case%20Study_Final.pdf
3. Action Against Hunger. Surveillance and Evaluation Team (SET) and Multi-Sectoral Emergency Team (MET): An integrated emergency response- South Sudan 2018. https://www.alnap.org/system/files/content/resource/files/main/FinalEvaluationReport%20_Sb2S_2018.pdf
4. Action Against Hunger. Annual progress report. 2014 https://actionagainsthunger.ca/cms/wp-content/uploads/2015/07/acf_annualprogressreport_2014.pdf
5. Adrianopoli, Marina, and Allan Mpairwe. WHO emergency nutrition response in South Sudan." Field Exchange 53 (2016): 69.
6. Ahn, E., Ouma, C., Loha, M., Dibaba, A., Dyment, W., Kim, J., Beck, N.S. and Park, T., 2020. Do we need to reconsider the CMAM admission and discharge criteria?; an analysis of CMAM data in South Sudan. BMC public health, 20(1), pp.1-10.
7. Amegovu AK, Mori J, Chewere T, , Muyima L, Jokudu S and Mawadri M. Nutrition Specific Interventions for Management of Malnutrition in Eastern Equatoria state, South Sudan – Challenges and Lessons for Non-Governmental Organizations https://clinical-nutrition.imedpub.com/nutrition-specific-interventions-for-management-of-malnutrition-in-eastern-equatoria-state-south-sudan-challenges-and-lessons-for.pdf
8. Alumai, J.B., 2020. The effectiveness of nutrition education for care takers at Al-Sabah children hospital in patient therapeutic feeding center, Juba South Sudan. Open Science Journal, 5(3).
9. Australian Aid. Australian Humanitarian Partnership. South Sudan Humanitarian Response Evaluation - Executive Summary. 2018 https://static1.squarespace.com/static/5ab0691e5417fc8a1ee9a417/t/5d22a6180b9e9c000191171d/1562551841799/AHP+South+Sudan+Evaluation+Final+Report+Exec+Summary.pdf
10. CDC, Concern, GOAL, IMC, and ACF. CMAM report: Development of CMAM Report – an online reporting system for CMAM programming Background, evaluations and lessons learnt. 2014. https://www.elrha.org/wp-content/uploads/2015/01/Appendix-2-MRP-project-write-up-FINAL.pdf
11. Concern Worldwide. South Sudan: Food Insecurity - 2015-2021 (ongoing- interim report) https://reliefweb.int/disaster/ce-2015-000183-ssd

12. Concern Worldwide. Adaptations in the management of child wasting in the context of COVID-19 in South Sudan. https://reliefweb.int/sites/reliefweb.int/files/resources/CMAM%20COVID%20Adaptations_South%20Sudan%20Case%20Study_Concern%20Worldwide_Final%20Feb%202021.pdf
13. Daniel T, Mekkawi T, Garelnabi H. Scaling up CMAM in protracted emergencies and low resource settings: experiences from Sudan. Field Exchange 2016;55:74.

Food Security and Nutrition Monitoring Report (FSNMS)

14. Round 14
15. Round 15
16. Round 16
17. Round 17
18. Round 18
19. Round 19
20. Round 20
21. Round 21
22. Round 22
23. Round 23
24. Round 24
25. Round 25
26. European Commission. Evaluation of DG ECHO's Response to the Humanitarian Crises in Sudan and South Sudan (2011-2015). Luxembourg: Publications Office of the European Union, 20172016. https://ec.europa.eu/echo/files/evaluation/2017/complet-report.pdf
27. Government of South Sudan: Community management of acute malnutrition (CMAM) guidelines. Juba: Ministry of Health, Government of South Sudan; 2017.
28. Government of South Sudan: Guidelines for inpatient management of severe acute malnutrition. Juba: Ministry of Health, Government of South Sudan; 2018.
29. Government of South Sudan, WFP, UNICEF, and FAO. Integrated Food & Nutrition Security Causal Analysis- Warrap and Northern Bahr el Ghazal. Juba: Ministry of Health 2017
30. Grellety E, Krause LK, Eldin MS, Porten K, Isanaka S. Comparison of weight-for-height and mid-upper arm circumference (MUAC) in a therapeutic feeding programme in South Sudan: is MUAC alone a sufficient criterion for admission of children at high risk of mortality?. Public health nutrition. 2015 Oct;18(14):2575-81.
31. Hailey, Peter, and Brenda Akwanyi. Strengthening nutrition humanitarian action: Supporting humanitarian cluster/sector coordination transition." Field Exchange 56 (2017): 42-46.

32. Kozuki N, Seni M, Sirat A, Abdullahi O, Adalbert MF, Biotteau M, Goldsmith A, Dalglish SL. Adapting acute malnutrition treatment protocols in emergency contexts: a qualitative study of national decision-making. Conflict and Health. 2020 Dec;14(1):1-2.
33. Kozuki N, Van Boetzelaer E, Zhou A, Tesfai C. Enabling treatment of severe acute malnutrition in the community: study of a simplified algorithm and tools in South Sudan. International Rescue Committee; 2018.
34. Kozuki N, Seni M, Sirat A, Abdullahi O, Adalbert MF, Biotteau M, Bailey J, Goldsmith A, Dalglish S. Factors affecting decision-making on use of combined/simplified acute malnutrition protocols in Niger, north-east Nigeria, Somalia and South Sudan. Field Exchange issue 60. 2019 Jan 7:38.
35. Kozuki N, Tesfai C, Zhou A, van Boetzelaer E. Can low-literate community health workers treat severe acute malnutrition? A study of simplified algorithm and tools in South Sudan. Field Exchange 59. 2019 Jan 27:30.
36. Laker M, Toose J. Nutrition programming in conflict settings: lessons from South Sudan. Field Exchange 2016;53:2
37. Laker M, Emmanuel S, Scott J. Scaling Up Nutrition (SUN) in Protracted Crisis: The South Sudan Civil Society Alliance (CSA) experience. Field Exchange 58. 2018 Jan 9:75.
38. Logue S. Tech RRT Assessment Adviser: Experiences from South Sudan, Mozambique, Iraq and Yemen. Field Exchange 56 (2017): 71-72.
39. Manyama IB. Partnership and accountability in the South Sudan Nutrition Cluster (2015-2017). Field Exchange 56. 2017 Dec 17:29-75.
40. Manyama IB. South Sudan Nutrition cluster 2017: famine lessons learnt. Field Exchange 56. 2018 Feb 1:59-61
41. Marron, Bethany, Pamela Onyo, Eunice N. Musyoki, Susan Were Adongo, and Jeanette Bailey. "ComPAS trial in South Sudan and Kenya: headline findings and experiences." Field Exchange issue 60 (2019): 19.
42. Mayai AT, Akol ZD, Garang JA, Mou AA, Anei TD, Akau SG. Food Security and Nutrition Vulnerability and Risk Analysis in Former Warrap and Northern Bahr el Ghazal States.
43. Mung'ou, Titus. "South Sudan nutrition: Overcoming the challenges of nutrition information systems." Field Exchange 53 (2016): 73.
44. Murphy M, Abebe K, O'Mahony S. Management of acute malnutrition in infants less than six months in a South Sudanese refugee population in Ethiopia. Field Exchange 2017;55:70.
45. Mwai, D. A cost analysis of community management of acute malnutrition (CMAM) program in South Sudan. Nairobi: University of Nairobi, Kenya. 2020
46. Okello FA. Assessment of the performance of the community based outpatient therapeutic program in Abyei administrative area, South Sudan. Doctoral dissertation, Uganda Christian University.

47. OCHA. Financial Tracking Service. Republic of South Sudan 2018 https://fts.unocha.org/appeals/646/projects?f%5B0%5D=destinationOrganizationIdName%3A3853%3AMedici%20con%20l%27Africa%20CUAMM&f%5B1%5D=destinationOrganizationIdName%3A8242%3APremi%C3%A8re%20Urgence%20Internationale and
48. OCHA. Financial Tracking Service. Republic of South Sudan 2019 https://fts.unocha.org/appeals/713/flows?f%5B0%5D=destinationClusterIdName%3A4508%3ANutrition
49. Save the Children. Innovations in Community Management of Acute Malnutrition (CMAM). 2018. https://www.ennonline.net/attachments/3021/Innovations-in-CMAM-FINAL_Updated-October-2018.pdf
50. South Sudan Nutrition Cluster. Cluster Performance Monitoring – Final report. 2015. https://www.nutritioncluster.net/sites/nutritioncluster.com/files/2020-01/SouthSudan-CCPM-Final-Draft-08-June-2015-under-SAG-review.pdf
51. Sessions, N. and Kopplow, R., 2018. Nutrition programming in Northern Bar el Ghazal, South Sudan: A time to reflect. Field Exchange 58, p.84.
52. South Sudan Nutrition Cluster. JULY—SEPTEMBER 2017 QUARTERLY BULLETIN: Summary. https://reliefweb.int/sites/reliefweb.int/files/resources/nutrition_cluster_quarterly_bulletinjuly-september_2017v3.pdf
53. South Sudan Nutrition Cluster. JULY—DECEMBER 2015 BULLETIN: Summary. https://www.humanitarianresponse.info/sites/www.humanitarianresponse.info/files/documents/files/nutrition_cluster_quarterly_bulletin_july-december_2015_0.pdf
54. United Nations Children’s Fund. Scaling-up care for children with severe acute malnutrition in South Sudan: Lessons learned from expanding quality services in a complex emergency context, New York: UNICEF; 2020
55. UNICEF: Treatment of wasting using simplified approaches: A rapid evidence review https://www.unicef.org/media/97006/file/Simplified-Aproaches-Rapid-Evidence-Review.pdf
56. UNICEF. Annual Report 2017: SOUTH SUDAN https://www.unicef.org/southsudan/media/406/file/UNICEF-South-Sudan-Annual-Report-2017.pdf
57. NICEF. Annual Report 2016: SOUTH SUDAN https://www.unicef.org/southsudan/media/411/file/UNICEF-South-Sudan-Annual-Report-2016.pdf
58. United Nations. SOUTH SUDAN- Humanitarian Response Plan 2015 https://reliefweb.int/sites/reliefweb.int/files/resources/SOUTH%20SUDAN%20HRP%202015_0.pdf

59. UNICEF: South Sudan Humanitarian Situation. Report No. 145. Reporting Period: May 2020 https://www.unicef.org/media/75756/file/South-Sudan-SitRep-May-2020.pdf
60. UNICEF South Sudan. South Sudan: Nutrition Programme Sectoral and OR+ (Thematic) Report Projects. 2019 https://open.unicef.org/sites/transparency/files/2020-06/South-Sudan-TP3-2018.pdf
61. UNICEF: Nutrition in South Sudan Briefing note. 2021 https://www.unicef.org/southsudan/media/7661/file/Nutrition%20Briefing%20Note_Jan-Mar%202021.pdf
62. UNICEF and Global Nutrition Cluster. South Sudan national CMAM overage workshop: 17-18 September 2014. Workshop report and national action plan. 2014 https://www.coverage-monitoring.org/wp-content/uploads/2014/12/South-Sudan-Workshop-Report-VF1.pdf
63. UNICEF; ACF, Nutrition surveys and SMART methodology in sub-Saharan Africa. Available at https://www.unicef.org/esa/sites/unicef.org.esa/files/2018-09/UNICEF-AAH-2016-SMART-Analytical-Report-Sub-Saharan-Africa.pdf
64. Van Boetzelaer, E., Zhou, A., Tesfai, C. and Kozuki, N., 2019. Performance of low-literate community health workers treating severe acute malnutrition in South Sudan. Maternal & child nutrition, 15, p.e12716.
65. Warille MA. Audit of care of severely acute malnourished children aged 6-59 months admitted at Alsabah Children Hospital-Juba (South Sudan) (Doctoral dissertation, University of Nairobi).
66. WFP. Country portfolio evaluation. South Sudan: An evaluation of WFP's Portfolio (2011 - 2016). Evaluation Report – Volume I Report number: OEV/2016/013 https://docs.wfp.org/api/documents/WFP-0000022545/download/
67. WFP and UNICEF: South Sudan: UNICEF and WFP Joint Nutrition Response Plan June 2015 — May 2016 https://www.unicef.org/southsudan/media/706/file/UNICEF-WFP-Joint-Nutrition-Response-Plan-South-Sudan-2015-2016.pdf
68. WFP and UNICEF: South Sudan: UNICEF and WFP Scale-Up Nutrition Plan One Year Report https://www.unicef.org/southsudan/media/721/file/UNICEF-WFP-Scale-Up-Nutrition-1Yr-Report-South-Sudan-2014-2015.pdf
69. World Vision International. Annual Report 2018. Our Promise to the Children of South Sudan https://www.wvi.org/sites/default/files/WVSS_Annual%20Report%202018_Apr-15_FINAL.pdf
